# New epigenetic players in stroke pathogenesis: From non-coding RNAs to exosomal non-coding RNAs

**DOI:** 10.1016/j.biopha.2021.111753

**Published:** 2021-05-25

**Authors:** Maryam Mahjoubin-Tehran, Samaneh Rezaei, Amin Jesmani, Nafise Birang, Korosh Morshedi, Hashem Khanbabaei, Haroon Khan, Ashkan Piranviseh, Majid Nejati, Michael Aschner, Hamed Mirzaei

**Affiliations:** a Student Research Committee, Mashhad University of Medical Sciences, Mashhad, Iran; b Department of Medical Biotechnology, Faculty of Medicine, Mashhad University of Medical Sciences, Mashhad, Iran; c Department of Neurosurgery, Shariati Hospital, Tehran University of Medical Sciences, Tehran, Iran; d Department of Physical Medicine and Rehabilitation, School of Medicine, Shiraz University of Medical Sciences, Shiraz, Iran; e School of Medicine, Kashan University of Medical Sciences, Kashan, Iran; f Medical Physics Department, School of Medicine, Kerman University of Medical Sciences, Kerman, Iran; g Department of Pharmacy, Abdul Wali Khan University, Mardan 23200, Pakistan; h Brain and Spinal Cord Injury Research Center, Tehran University of Medical Sciences, Tehran, Iran; i Anatomical Sciences Research Center, Institute for Basic Sciences, Kashan University of Medical Sciences, Kashan, Iran; j Department of Molecular Pharmacology, Albert Einstein College of Medicine, Bronx, NY 10463, USA; k Research Center for Biochemistry and Nutrition in Metabolic Diseases, Institute for Basic Sciences, Kashan University of Medical Sciences, Kashan, Iran

**Keywords:** Stroke, Long non-coding RNAs, MicroRNAs, Pathogenesis

## Abstract

Non-coding RNAs (ncRNAs) have critical role in the pathophysiology as well as recovery after ischemic stroke. ncRNAs, particularly microRNAs, and the long non-coding RNAs (lncRNAs) are critical for angiogenesis and neuroprotection, and they have been suggested to be therapeutic, diagnostic and prognostic tools in cerebrovascular diseases, including stroke. Moreover, exosomes have been considered as nanocarriers capable of transferring various cargos, such as lncRNAs and miRNAs to recipient cells, with prominent inter-cellular roles in the mediation of neuro-restorative events following strokes and neural injuries. In this review, we summarize the pathogenic role of ncRNAs and exosomal ncRNAs in the stroke.

## 1. Introduction

Stroke is a debilitating disease, and the 2nd main cause of global mortality. As stated by the WHO, almost 15 million people experience strokes worldwide, of which 5.5 million die each year [1]. Stroke prevalence, particularly in developing countries continues to grow, causing major social and economic burdens [2].

LncRNAs have key functions in various disorders. Numerous abnormally expressed lncRNAs are detected in patients with ischemic stroke or animal models of ischemia [3]. LncRNAs play a role in angiogenesis, inflammation, cell apoptosis and cell death. MicroRNAs (miRNAs) are a group of small non-coding RNAs [4]. Following stroke, miRNAs affect various pathophysiological mechanisms, such as hematopoiesis, proliferation or rapid growth, immune function, depression as well as metabolism [5]. Indeed, many investigations have corroborated the aberrant expression of miRNAs in stroke [5,6]. These studies establish miRNAs as key mediators in pathological and pathogenic features of ischemic stroke.

Exosomes are extra-cellular vesicles formed by fusion of multi-vesicular bodies with plasma membrane with subsequent release from cells. Exosomes are 30–100 nm in size, and express several cell-surface markers such as CD-63 and Alix. They are characterized by 1.13–1.19 g/mL density in sucrose and thus can be sedimented at 100,000 g [7]. Non-coding RNAs are released from cells in exosomes. Exosomal cargos, such as cellular proteins, non-coding RNAs(lncRNAs and mRNAs)and lipids act as mediators of inter-cellular cross-talk between recipient and effector cells [8]. Additionally, cargo preservation from degradation and transport across the blood-brain barrier (BBB) into the systemic circulation renders exosomes as potential disease biomarkers [9]. Exosomes contribute to neuro-inflammatory stress response as well as neurodegenerative diseases such as Parkinson’s disease (PD), stroke, Alzheimer ‘s disease (AD) and Schizophrenia [10]. Herein, we highlight the pathogenic role of non-coding RNAs in the stroke. Moreover, we summarize the role of exosomal non-coding RNAs in the stroke pathogenesis.

## 2. Biogenesis of lncRNAs

**Fig. 1.**
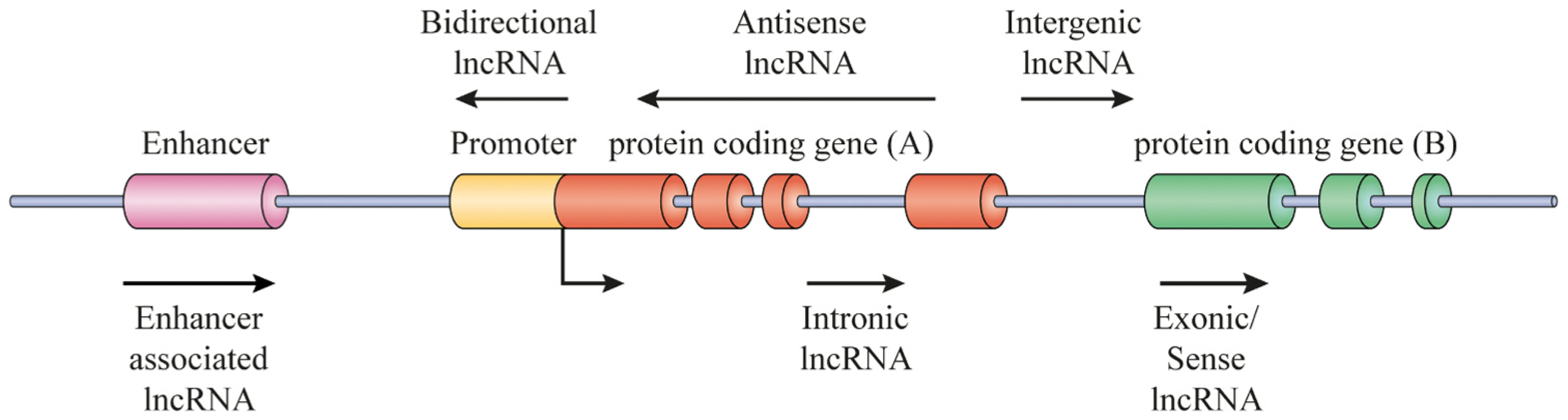
Biogenesis of lncRNAs. LncRNA transcripts are classified based on their genomic location in relation to the closest gene including antisense lncRNAs, sense lncRNAs, intronic lncRNAs, enhancer lncRNAs and intergenic lncRNAs.

LncRNAs are important mediators of health and disease [11–17]. Several classes of lncRNAs exist, which are transcribed from several DNA elements including intergenic regions, promoters, and enhancers in eukaryotes [18] (Fig. 1). A variety of mechanisms are involved in the biogenesis of lncRNAs. including generation of mature ends by ribonuclease P (RNaseP) cleavage, creation of small nucleolar RNA (snoRNA), the formation of protein (snoRNP) complex caps at their ends, as well as creation of circular structures[19,20]. During biogenesis of lncRNAs, sub-nuclear structures, termed “paraspeckles”, are found in close proximity to specific lncRNAs [21]. Four specific proteins (PSPs) are required for paraspeckle formation [21,22]. The exact mechanism of synthesis and regulation of lncRNAs has yet to be detailed. Techniques, such as cross linking immunoprecipitation (CLIP), ChIRP-Seq (Chromatin Isolation via RNA purification), phylogenetic lineage tracing, RNA structure mapping, ribosome profiling, genetic screens, and genome engineering through CRISPR [23–25], are likely to help in this task.

## 3. lncRNAs and stroke

**Fig. 2.**
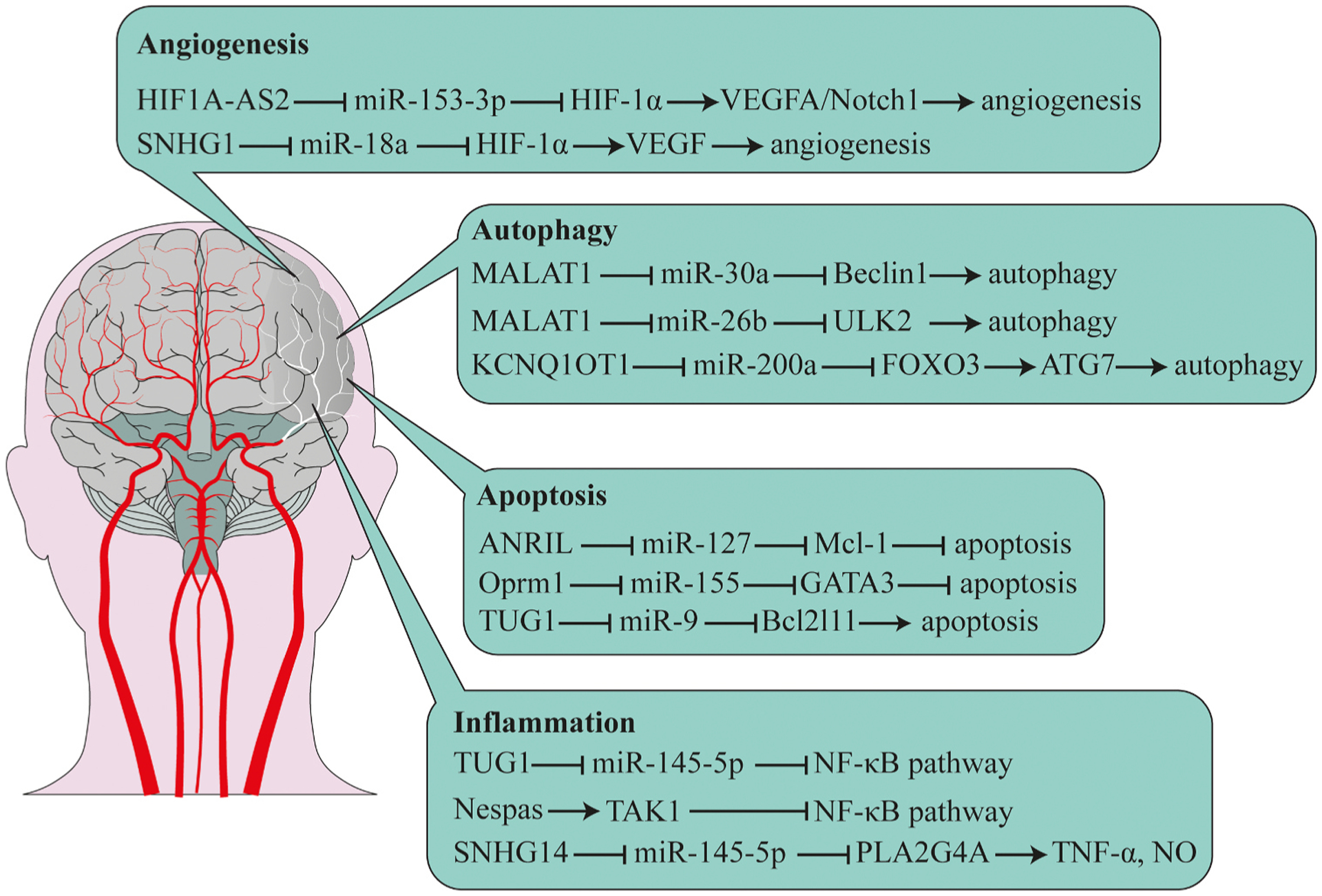
Different lncRNAs involved in stroke pathogenesis. This figure is adapted from [82].

Long non-coding RNAs play a significant role in ischemic stroke. Numerous aberrantly expressed lncRNAs have been identified in ischemic stroke samples of human or animal models [26,27]. Among them, anti-sense non-coding RNA in INK4 locus (ANRIL), MALAT1,H19, N1LR, the maternally expressed gene 3 (MEG3), CaMK2D-associated transcript 1 (C2dat1), taurine up-regulated gene 1 (TUG1),small nucleolar RNA host gene 14 (SNHG14), and Fos downstream transcript (FosDT) were shown to affect angiogenesis, cell death, cell apoptosis, as well as inflammation in the course of ischemic stroke (Table 1, Fig. 2).

### 3.1. Angiogenesis

One of the first identified lncRNAs is MALAT1, which promotes cancer proliferation and metastasis via gene expression and alternative splicing [28–31]. MALAT1 is a stable and conserved lncRNA (~7 kb ([32], and is expressed in vascular endothelial cells, skeletal muscle, cardiomyocytes. MALAT1 thought to be involved in angiogenesis and pathological myogenesis [33–36]. In addition, high expression of MALAT1 is inherent to cell nucleus speckles, a pre-mRNA processing domain with a role in gene expression regulation or organization[37]. MALAT1 can also affect pre-mRNA splicing by interacting with splicing factors [38], and in cancers, MALAT1 expression is upregulated by HIF-1αor hypoxia [39,40].

In animal stroke models, such as middle cerebral artery occlusion (MCAO) and oxygen-glucose deprivation (OGD), MALAT1 expression is significantly increased (6.05-fold) [26]. MALAT1knockout mouse leads to a larger size brain infarct, reduced sensorimotor function, and decreased neurological scores after MCAO [41]. Knockout of MALAT1is also associated with reduced vascular growth mice retinas [42], consistent with studies on angiogenesis in experimental cancer models [42–44]. In the post-ischemic phase, cerebral vasculature has an important role in the recovery outcomes. Thus, increasing expression of MALAT1 in ischemic stroke affords protection. Further, MALAT1 knockout is characterized by increased expression of Bim, apro-apoptotic factor, pro-inflammatory cytokines, inter-leukin 6 (IL-6), E-selectin, as well as monocyte chemotactic protein-1 (MCP-1),both in ischemic murine brain and brain microvascular endothelial cells (BMECs) [41]. These findings suggest that MALAT1 has protective effect on cerebral ischemic injuries by inhibiting inflammation and endothelial cell death.

### 3.2. Inflammation

ANRIL, an anti-sense RNAs co-clustered with the p15/CDKN2B-p16/CDKN2A-p14/ARF locus in chromosome 9p21, is involved in cancers and cardiovascular diseases [45–47]. ANRIL has several splice variants with a length of ~3.9 kb [48]. In cancers, ANRIL activates by factor-1α and c-Myc which are hypoxia-inducible [49,50]. ANRIL can bind to PRC1/2 and mediates silence gene expression of INK4b-ARF-INK4a locus [51]. The enhanced expression of ANRIL is correlated with coronary artery disease, atherosclerosis, as well as stroke [52–55]. ANRIL expression, associated to variants of chromosome 9p21.3, has been suggested as a novel genetic marker for stroke [56]. Its expression is increased in rat cerebral infarcted cortex [57], resulting in activation of IκB/NF-κB pathways and vascular-endothelial growth factor (VEGF)/-VEGF receptor 1 (FLT-1) [57], thus promoting inflammation and angiogenesis. Binding the VEGF to FLT-1 acts as a stimulus for fetal angiogenesis, as well as maintaining endothelial function in adult animals [58]. NF-κB dissociates from IκB in stresses e.g. oxidative stress (OS), cytokines, ultra-violet irradiation, and viral or bacterial antigens. NF-κB translocates to the nucleus and modulates adaptive immune response genes [59]. By activating the VEGF/FLT-1 as well as IκB/NF-κB pathways, ANRIL may significantly contribute to pro-inflammation and angiogenesis.

In addition, caspase recruitment domain (CARD) family member 8, referred to as TUCAN/CARDINAL, is another ANRIL target [60]. rs2043211, an SNP in CARD8 decreases CARD8 expression, and is associated with reduced risks of ischemic stroke [61]. Furthermore, increase or decrease in ANRIL stimulates or inhibits CARD8expression in HepG2 cells, respectively [62]. ANRIL activation suppresses NF-κB by activating CARD8; therefore, likely inhibiting inflammation. Overall, the increased ANRIL levels enhance angiogenesis via VEGF/FLT-1 pathway, and regulate inflammation through NF-κB pathway in ischemic stroke.

### 3.3. Autophagy

LncRNA-H19 is a 2.3 kb conserved RNA coded by the H19 gene and expressed only in the maternal allele [63]. H19 control growth and development in the embryo [64]. At the first stage (6–8 weeks gestation) of embryonal development, both H19 alleles (paternal and maternal) are expressed. After 10-weeks of gestation, only the maternal H19 alleles are expressed [65]. By targeting another imprinted gene, Igf2, H19 can gain function in controlling the embryo’s growth [66]. The hypermethylation of the H19 promoter and methylation of the 3′ side of H19 in a specific allele is related to the change in H19 expression [65]. Indeed, in pathological conditions, for example, oxidative stress and cancer, the expression of H19 is upregulated [67–69]. H19 levels increased in stroke subjects compared to healthy subjects [70] affording diagnostic tool for ischemic stroke [70]. Up-regulation of H19 expression has been also been shown in OGD/reoxygenation in SH-SY5Y cells and ischemia/reperfusion rat brain [71]. Variation in H19 alleles, such as rs217727, has been shown to increase ischemic stroke risk [71]. Additional investigations showed that inhibiting H19 protects SH-SY5Y cells from autophagy and cell death induced by OGD/R. The dual specificity phosphatase 5 (DUSP5)-ERK1/2axis has a role in pro-autophagy impact on H19. DUSP5, a protein kinase phosphatase activated by mitogens, inhibits the ERK1/2 pathway and autophagy [71–73]. Increased levels of H19 suppress DUSP5, and therefore activate autophagy and ERK1/2.

### 3.4. Apoptosis

N1LR, is a lncRNA, located on chromosome 9, with 1.8 kb length; its sequence has overlap with 5′-UTR of Nck1 gene sequence. To date, only one study examined N1LR’s function [74]. N1LR is expressed in cerebral ischemia/reperfusion rat model [75]. Decreased N1LR expression affects infarct volume. Reduction of N1LR expression is inherent toN2a cells undergoing OGD/R. Upregulation of N1LR is associated with a reduction in OGD/R-induced apoptosis in N2a cells by inhibiting the p53 activation [75]. Analysis of the genome location as well as RACE assay has shown overlapping between the N1LR sequences with 5′-UTR of *Nck1*. The *Nck1* gene encodes a protein that plays an important role in glucose tolerance, insulin signaling, and cellular remodeling. *Nck1* is increases in the brain of ischemic rats. Furthermore, the genetic ablation of lncRNA-N1LR increases *Nck1* expression. However, increasing of lncRNA-N1LR expression did not impact*Nck1*expression [74,76].

FosDT, a 604nt lncRNA, also termed MRAK159688, has an overlapping sequence with the down-stream of Fos [77]. The expression of FosDT is up-regulated (about 13 folds compared to the control) in the acute period of MCAO after focal ischemia [78]. Increased FosDT expression is associated with neurological dysfunction and post-stroke brain damage. Suppressing FosDT causes a reduction in infarct volume and improved recovery of post ischemia motor function in comparison with controls [78]. Bio-informatics analyses has shown that FosDT and *Fos* are congenic on chromosome 6q31 in rats [78]. *Fos* rapidly increased after brain injury [79]. The correlation between increased *Fos* with increased FosDT suggests transcriptional and/or regulatory interactions between them. FosDT attaches to the chromatin modifying proteins (CMPs) including Sin3a as well as co-repressors of transcription factor REST (coREST). These proteins are co-repressors for the transcription factor repressor element-1 silencing transcription factor (REST) [80]. In fact, REST represses neuronal function such as synaptic transmission and neural differentiation [81]. Furthermore, REST forms a complex consisting of Sin3a (REST-coREST-Sin3a) as well as coREST in rats with transient focal ischemia. This complex inhibits the expression of downstream genes such as NF-κB2, GluR2 and N-methyl-D-aspartate 1 and increases ischemic brain damages[78].

## 4. MicroRNA biogenesis

MicroRNAs are short (~22-nucleotide) non-coding RNAs which silence gene expression post-transcriptionally. The first miRNA has been identified in *Caenorhabditis elegans* in 1993 [88]; but the regulatory function of miRNAs was first recognized in 2001 [89–91]. miRNA binds to the target mRNA degrades it or blocks translation [92]. RNA polymerase II (Pol II) the most of miRNA genes in the nucleus to primary miRNAs (pri-miRNAs). Next, capping, splicing and polyadenylation are carried out by pri-miRNAs [93]. Most of the miRNAs are transcribed from dedicated miRNA gene loci and only 30% of miRNAs are expressed from introns. A single pri-miRNA may produce one or several miRNAs.

Long pri-miRNA is cleaved via Micro-processor consisting of DRO-SHA, an RNase III enzyme, as well as the respective co-factor; namely, DiGeorge syndrome critical region 8 (DGCR8) [94,95]. Two RNase III domains have been identified in DROSHA; each domain processes one strand of the dsRNA to cleave almost 60–70-nucleotide hairpin-shaped precursor miRNAs (pre-miRNAs) [94–97]. Microprocessor identifies the single-stranded RNA (ssRNA)–stem junction along with distance from the terminal loop. Therefore, it cleaves dsRNA from the junction with the flanking ssRNA and pre-miRNAs in the shape of the hairpin produce with a 1 or 2 nucleotide overhang at the 3′ end (group I and II miRNAs, respectively) [98–101]. Though the core components of DROSHA as well as DGCR8 have been regarded as essential for nearly all miRNA biogenesis, and micro-processor activities may be re-constituted with the recombinant DGCR8 and DROSHA proteins in vitro [95,96],

A variety of factors play important roles in processing of pri-miRNA (more details are illustrated later). Pre-miRNAs are exported to the cytoplasm through exportin 5 (XPO5)[102–104]. In the cytoplasm, DICER1 (RNase III enzyme) further processes pre-miRNAs [105]. In addition, DICER1 attachment to the end of pre-miRNA and a-symmetrical cleavage of dsRNA stem. DICER1cleavages near the terminal loop and creates a mature miRNA duplex; while, 2-nucleotide are over hanged in the 3′ end [106]. Hence, DICER1 correlates with the transactivation responsive RNA-binding protein (TRBP),TARBP2, for binding to dsRNA [107]. TRBP increases the DICER1 cleavage fidelity of pre-miRNAs in a structure-dependent manner. TRBP also changes the selection of guide-strand of miRNA by promotion the formation of isomiRNAs, which are one nucleotide longer than the usual miRNAs; but TRBP is not essential for processing of pre-miRNA by DICER1 [108,109]. Moreover, TRBP physically links DICER1 and Argonaute proteins, including AGO2, AGO1, AGO4 and AGO3 in order to involve in formation of a miRNA-induced silencing complex (miRISC) [107]. The guide strand of the miRNA through an Argonaute protein retains in the miRISC. Then miRNA guides the complex to target mRNAs and post-transcriptionally silence gene expression. Such a condition is found in cytoplasmic foci, processing bodies (P-bodies) induced by mRNA silencing; however, P-bodies are not essential for gene silencing mediated by miRNA [110–112].

## 5. MicroRNAs and stroke

Expression of miRNAs after stroke has an important role in the disease. For example, three miRNAs including miR-497, miRNA 21 and miR-99a have been shown to attenuate ischemic volumes and save the neuronal cells from apoptosis, preserving neurological functions [113–115]. In addition, overexpression of miR-let-7c-5p and miR-424 showed reduced activation of microglia in cerebral ischemia [116]. Up-regulation of miR-103, miR-103, miR-132 as well as miR-126 diminish neurobehavioral as well as neuropathological alterations in hemorrhagic stroke. These effects occur by protection of BBB integrity, attenuating neuroinflammation and decreasing neuronal apoptosis [117–119]. Moreover, neurogenesis and angiogenesis in mouse brain is ameliorated by up-regulation of miR-210, promoting repair [120]. However, miRNA can also have a negative role. For instance, Liu et al. reported that miR-155 silencing increases migration, proliferation, and angiogenesis due to reduced cellular apoptosis, and production of reactive oxygen species (ROS) [121]. In agreement, in hemorrhagic stroke, miR-27b silencing has been shown to mitigate neurological loss by reducing cell death and repressing neuro-inflammation. Additionally, miR-124 and miR-155 have been shown to play a role in macrophage polarization [122]. Furthermore, miRNAs have an opposite effect in modulation of the synaptic plasticity. For example, miR-134 has been shown to improve the remodeling of neuronal structures through translational repression of Limk1-mRNA,a protein kinase with a role in dendritic spine development [123].

miR-212-5p is involved in ferroptotic neuronal death in traumatic brain injury (TBI)murine model. Overexpression of miR-212-5p attenuated ferroptosis while downregulation of miR-212-5p promoted ferroptotic cell death partially by targeting prostaglandin-endoperoxide synthase-2 in HT-22 and Neuro-2a cell lines. In addition, administration of miR-212-5p in controlled cortical impact mice significantly improved learning and spatial memory [124].

Compared with non-DM stroke mice, T2DM-stroke mice exhibit significantly decreased serum and brain tissue miR-126 expression. Endothelial cells and EC-Exo contain high levels of miR-126 compared with other cell types or exosomes derived from other types of cells, respectively (smooth muscle cells, astrocytes, and marrow stromal cells). EC-Exo treatment of T2DM-stroke mice significantly improves neurological and cognitive function, increases axon density, myelin density, vascular density, arterial diameter, as well as induces M2 macrophage polarization in the ischemic boundary zone [125].

### 5.1. Inflammation

Xie et al. reported on the ability of miR-181a to promote the cellular survival in vitro through repressing inflammation processes in macrophages as well as monocytes [126]. However, Moon has shown that miR-181a silencing attenuated neuronal apoptosis induced by forebrain ischemia [127].

Inflammation is an intricate response after injury and promotes repair process [128]. Enhanced immune responses may be detrimental [129], and can be an important determinant of stroke prognosis [117, 130–132]. Both hemorrhagic and ischemic stroke trigger microglial activation and inflammatory factors release, including TNF-α [133,134], promoting brain injury [135–137]. Moreover, T-lymphocytes, natural killer cells, polymorpho-nuclear leukocytes and mononuclear phagocytes produce and release several peripherally-derived cytokines which participate in neuroinflammation after stroke [138,139].

Numerous genes function to regulate neuroinflammation. These genes are targeted by miRNAs [140–142]. Lentiviral over-expression of miR-424 diminishes brain injuries following the ischemic stroke by repressing microglial activity [116]. miR-let-7c-5p has a neuro-protective effect against neuroinflammation after ischemic stroke, preventing the translational repression of caspase-3 and microglia activation [143]. miR-124 is known as ‘brain-specific miRNA’ because it is expressed particularly in the central nervous system (CNS) [144,145]. Laterza et al. demonstrated that miR-124 is overexpressed in plasma following the brain injuries caused by MCAO [144]. Additionally, this microRNA suppressed the CCAAT/enhancer-binding protein alpha (C/EBP-α) and PU.1 directly as its down-stream element, resulting in microglia quiescence. Furthermore, miR-124 inhibited experimental autoimmune encephalomyelitis (EAE) by macrophage deactivation.

Toll-like receptors (TLRs) have been shown to have major functions in neuro-inflammation following stroke [146–148]. Zhang et al. reported that miR-181c inhibited expressing TLR4 via attachment to 3′UTR of its gene, thus reducing levels of the nuclear factor kappa light chain enhancer of the activated B cells (NF-κB) as well as generation of the down-stream pro-inflammatory factors [149]. Furthermore, up-regulation of miR132 in mice hemorrhagic stroke leads to a better prognosis in comparison to controls. In addition, over-expression of miR132 represses activated microglia and pro-inflammatory cytokine production [119]. Yuan and colleagues in an experimental model of ICH have shown that miR-367 reduced IRAK4 levels by direct binding to its 3′-UTR. MiR-367 also inhibits NF-κB activation and synthesis of its downstream pro-inflammatory elements. Another study has shown that miR-223 improves the neurological functions by down-regulating NLRP3 as well as inflammation inhibition via caspase-1 and IL-1beta [150].

MiRNAs have key roles in anti-inflammatory impact in brain due to the regulation of microglia and microphage polarization. MiR-155, by targeting M2-associated genes, promotes the M1 phenotype. Indeed, this microRNA targets different genes related to the M2 phenotype that mitigate production of M2-induced proinflammatory factors such as IL13Rα1, CD206 and IL-10 and Arg-1 [151,152].

### 5.2. Apoptosis

Apoptosis is energy-dependent and also known as programmed cell death [153]. Apoptosis has a key role in physiological metabolism, growth, and development. [154,155]. However, uncontrolled apoptosis may lead to different disorders such as cancers, Alzheimer’s disease as well as stroke [156,157]. Apoptosis is triggered by either internal or external pathways. External pathways occur surface death receptors activation. These receptors include Fas and TNF-related apoptosis like tumor necrosis factor (TNF)-α and ligand receptors, whereas interior pathways are associated with mitochondrial signaling pathways [158, 159]. Following stroke, a mass influx of Ca^2+^ into the cell is triggered, resulting in apoptosis-inducing factor (AIF) release or mitochondrial cytochrome c (Cytc) [160]. Binding Cytc to the apoptotic protease-activating factor-1 as well as procaspase-9 forms an apoptosome, in turn activating caspase-9. Eventually, caspase-3 leads to damages to nDNA as well as the ensuing cell death. In addition, AIF translocates to the nucleus, causing a large-scale (50 kb) DNA-fragmentation and cell death mediated by caspase activation [161].

miRNAs regulate post-stroke neuronal survival through regulation of the level of target genes [162,163]. miR-298 has been overexpressed in blood and brain specimens in experimental models of cerebral ischemia as well as ICH models [164]. An anti-apoptotic factor is miR-21. Buller et al. studied the levels of miR-21 expression both in vivo and in vitro. Levels of this microRNA increased following ischemic stroke secondary to decreased Fas ligand (FasL) G, a cell death-inducing ligand [115]. MiR-155 is responsible for regulating \cellular apoptosis via modulation of caspase-3 expression. Knocking down miR-155 decreased apoptosis in brain microvessel endothelial cells [121]. Moreover, miR-99a has been shown to prevent pro-caspase-3 activation and caspase-3 expression and decreased neuronal apoptosis following ischemic stroke. In addition, miR-99a mitigated neuronal injury after the cerebral Ischemia/Reperfusion (I/R), via cell cycle as well as the cellular apoptosis regulation. This suggests miR-99a as a novel treatment factor targeting neuronal cell cycle re-entry after ischemic stroke [114]. Additionally, the bcl-2 family contributes to apoptosis modulation. MiR-106b-5p, miR-181a, miR-497, miR-384-5p and miR134 increase apoptosis by reducing the levels of bcl-2 proteins [113,126,127,165,166].

MiR-132 has been shown to reduce neuronal mortality in ICH mice, following hemorrhagic stroke. Ip-regulation of miR-132 caused decreased likelihood of neurological deficits [119]. Lentivirus induced up-regulation of miR-126 was shown to be protective in ICH by triggering anti-apoptotic mediators secondary to reduced levels of caspase-3 [118]. Increased miR-103-3p expression in an experimental model of the sub-arachnoid hemorrhage associated with decreased levels of caveolin-1 has also been shown [167].

### 5.3. Oxidative stress

OS contributes to the pathogenesis of many disorders. It represents over-balance of pro-oxidants (ROS/RNS) and or deficiencies in the antioxidant systems in the cells [168–170]. Hence, free-radicals as well as ROS generation in the course of the stroke cause N-methyl-D-aspartic acid (NMDA) glutamate receptors [171], mitochondrial impairment, excessing Ca^2+^ [172–174], and activation of neuronal nitric oxide synthase (nNOS) [175]. Detoxifying enzymes and antioxidants include superoxide dismutase (SOD), glutathione reductase, glutathione-S-transferase and glutathione peroxidase, which sustain redox homeostasis [176,177]. Erythroid-2-related factor-2 (Nrf2) plays a neuroprotective role against brain damage after stroke, hydrogen peroxide (H_2_O_2_) exposure, glutamate excitotoxicity and Ca^2+^ overload [178].

miRNAs modulate Nrf2 mRNA levels [179]. Following ischemic stroke, miR-93 suppresses Nrf2 and hemeoxygenase-1 (HO-1) [180]. During cerebral ischemia, miR-424 reduces infarct volume by decreasing ROS levels in cortex and elevating manganese SOD (MnSOD) and extra-cellular SOD. miR-424 has also been shown to decrease H_2_O_2_ induced injuries in the neuronal cultures, by increasing cell viability as well as MnSOD activities, and decrease levels of lactate dehydrogenase leakage and malondialdehyde [181]. Moreover, miR-23a-3p and miR-106b-5p may have a neuro-protective impact against the post-ischemic oxidative injuries via overexpressing MnSOD [182,183]. Additionally, miR-145 suppresses SOD2 following ischemic stroke [184]. Xu et al. reported suppression of miR-27b reduces brain injury and results in overexpression of Hmox1, Nrf2, Nqo1 as well as SOD1-following ICH through Nrf2/ARE pathway [185]. Nonetheless, few investigations focused on the anti-oxidative activity of miRNAs in the hemorrhagic stroke. More studies are needed to investigate the contribution of the miRNAs in the hemorrhagic stroke.

### 5.4. Angiogenesis

Zhang et al. explored structural modifications following stroke, reporting increased vascular volumes from 3% to 6% at 90th days after stroke [186]. Angiogenesis regulator miRNAs is a possible therapeutic target in ischemic stroke [187]. MiR-210 has a significant function in angiogenesis following cerebral ischemia, partially by increasing vascular endothelial growth factor (VEGF) expression. In vitro, under hypoxic conditions, miR-210 mediates tube formation and migration of vascular endothelial cells [120]. Ma and colleagues reported that in a rat model of ICH, miR-129-5p represses the HMGB1-RAGE signaling pathway and consequently re-vascularization [188]. Up-regulation of miR126 is protective against ICH, increasing angiogenesis by elevating VEGF-A protein levels [160]. Hence, improved angiogenesis may represent a therapeutic modality for stroke through the pharmacological regulation of miRNAs.

Neurotrophic agents, representing small polypeptide molecules, have a key part in cell differentiation, proliferation, development and migration of the nervous system. Earlier investigations indicated that neuro-trophic agents such as brain derived neuro-trophic factor (BDNF), nerve growth factor, ciliary neurotrophic factor, insulin-like growth factor-1 (IGF-1) and glial-derived neurotrophic factor mitigate neuronal death as well as the brain lesions [189,190].

**Fig. 3.**
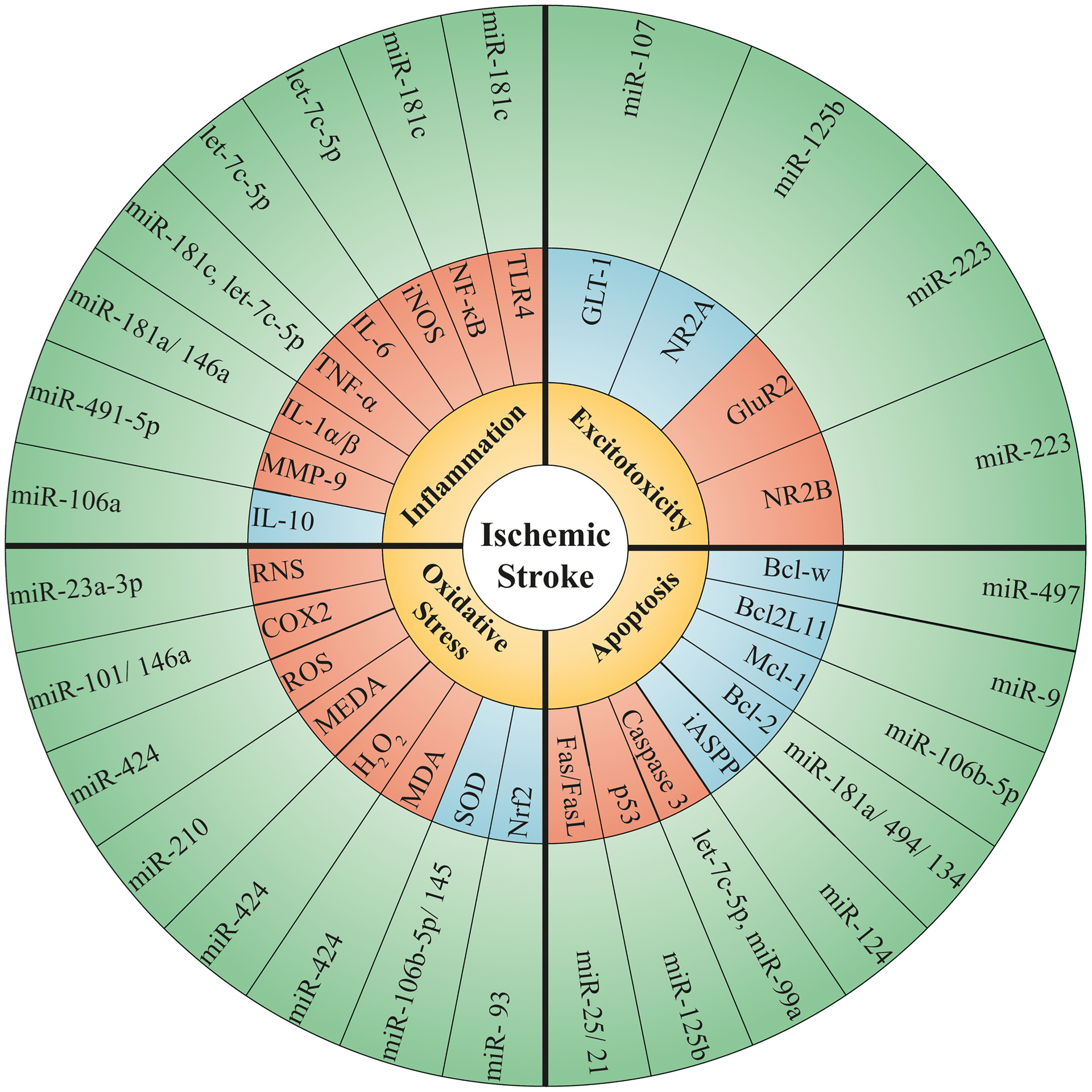
Various microRNAs involved in stroke pathogenesis.

miR-Let7f has been shown in an experimental model of cerebral ischemia to support IGF-1-like neuroprotection [190]. Additionally, suppression of miR-134 diminishes ischemic damages by promoting Bcl-2as well as BDNF expression. Moreover, miR-30-5p and miR-107 modulates BDNF expression [191]. In addition, neural precursor cells (NPCs) and endogenous neural stem cells (NSCs) can be switched on and migrate toward the injured location[192]. In this regard, miR-21 can modulate NPC’s activity via Wnt and transforming growth factor (TGF)-β signaling pathways. Furthermore, miR-34a modulates NPC proliferation negatively following cerebral ischemia [193]. Based on studies in type-2 diabetic mice following stroke, miR-126 may also promote the neuro-restorative function induced with umbilical cord blood cells [194]. Table 1 and Fig. 3 listed different miRNAs involved in stroke pathogenesis (Table 2).

## 6. Exosome biogenesis

Exosomes are largely conserved amongst eukaryotic organisms [207]. Exosomes originate via an endocytic route, and they are generated by the inward budding of plasma membranes [208].

Exosomes can be derived from stem cells isolated from different cell sources such as mesenchymal stem cells, endothelial progenitor cells, and fibroblasts.The mesenchymal stem cells (MSCs) are the most commonly used on research which have usually been isolated from synovium, bone marrow, and adipose tissue. The three major categories of extracellular vesicles are (a) apoptotic bodies, (b) microparticles or microvesicles, and (c) exosomes or nanovesicles [209].

Exosomes consist of the conserved proteins such as CD63 (membrane-related proteins like LAMP-3), CD9, Alix, CD81, as well as tumor susceptibility gene 101 protein [210], and the tissue- or cell-type-specific proteins, which indicate their cellular sources[211]. Sphingomyelin, ceramide and cholesterol are used to enrich exosome membranes [212]. On the other hand, exosomes consist of several biologically active molecules like proteins, deoxyribonucleic acids, ribonucleic acids (RNAs), microRNAs (miRs) and lipids [213]. Exosomal miRs or exosomal engineered miRs function in regulating the progression of various diseases, such as cancers, cardiovascular disease, and stroke [214–216].

Exosomal intercellular communications are mediated by bio-active molecules and are capable of targeting certain types of cells and modifying their target cell functions by delivering lipids, nucleic acids and proteins [217]. The majority of proteins in exosomes derive from the parent cell membranes, Golgi and the cytosol but seldom from endoplasmic reticulum or mitochondria [207]. Hence, cytosolic proteins will remain in exosomes and the ones derived from the plasma membranes will be kept in the vesicle membrane and maintain a similar topology of the cells with the potent contribution to sequestering the soluble ligands [218].

Exosomal proteins have a role in the antigen presentation, cell structure and motility as well as cell adhesion. Moreover, they modulate stress and contribute to transcription, protein synthesis, membrane fusion and trafficking [219]. Several functional impacts of exosomes could be ascribed to RNA and miR content transfer [217]. miRs and RNAs have been proposed as the most related cargo in the exosomes with regard to their abilities of a little number of molecules for influencing diverse proteins or enzymes in 1 or more cellular pathways in the targeted cells [220].

## 7. Exosomal microRNAs and stroke

**Fig. 4.**
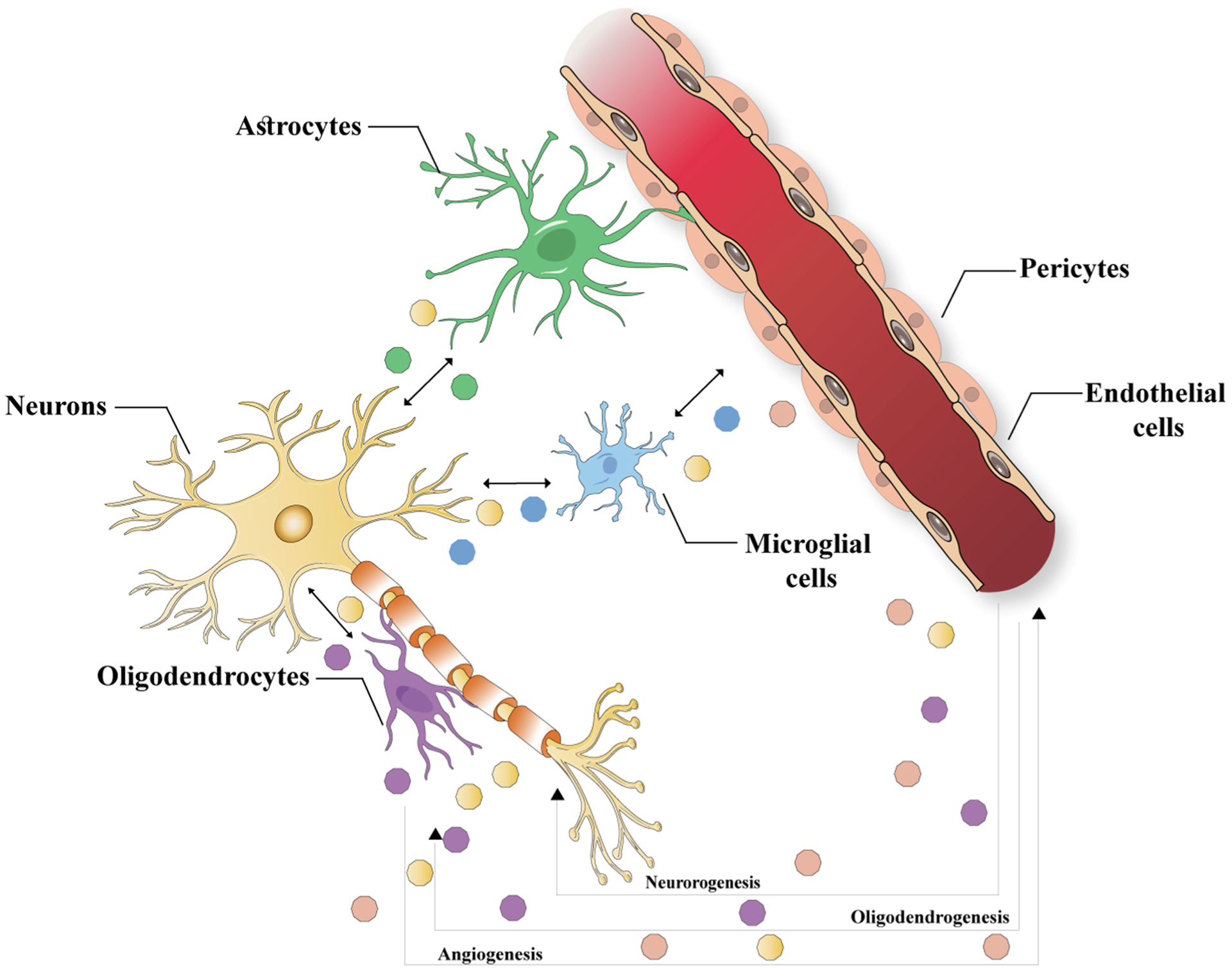
Potent exosome mediated inter-cellular communications in the brain re-modeling following the strokes. As seen, the exosomes transfer RNAs as well as proteins for influencing angiogenesis, oligo-dendrogenesis, EVs, extra-cellular vesicles and neurogenesis.

The ever-increasing information suggested the involvement of the exosome-mediated inter-cellular communication in the brain re-modeling via transfer of the cargo from the source cells to the targeted cells (Fig. 4) [221]. It is possible to isolate the exosomes from bio-fluids such as CSF and from the supernatant of the cells that have been cultured in the exosome-free medium via centrifugation and the other procedures [222]. Exosomes are commonly enriched with tetra-spanin proteins (CD81& CD63), the regulator of the endosomal trafficking Alix, as well as chaperone protein HSP70, though exosomes’ volume is variable according to the cell origin and pathological and physiological conditions [222,223]. Analyses of the proteomic and RNA analyses also showed that exosomes are the carriers of cargoes of proteins, RNAs and lipids such as miRNAs and mRNAs [222,223]. Nonetheless, there is insufficient information on the loading of biological substances into the individual exosomes. In fact, each brain cell releases exosomes [222,224, 225]. In comparison to exosomes separated from the wild type mice brains, exosomes from brains of transgenic mice overexpress human amyloid-β (Aβ) precursor protein (APP) [224]. In addition, the full-length APP cleavage via β-secretase takes place into the endosomes that is one of the fractions of Aβ peptides sorted for multi-vesicular bodies, and release of such Aβ peptides is seen in conjunction with exosomes [226]. Furthermore, exosomes isolated from the pre-frontal cortices of the cases suffering from schizophrenia and bi-polar disorder indicated diverse profiles of the exosomal miRNAs in comparison to the exosomes from control brains [225]. Collectively, these studies suggest that exosomes released by both human and mouse brains under disease conditions change profiles of exosomal cargo proteins and miRNAs and that exosomes enriched with neurotoxic C-terminal fragments of APP may contribute to the spread of Aβ peptides to the brain. Even though in vivo investigations could not specify the cellular source of the exosomes, information obtained from cultured cells suggested that exosomes released by neurons as well as the astrocytes consist of Aβ peptides [226,227].

The ever-increasing information also demonstrated miRNAs are the essential modulators in ICH [228,229]. Exosomal miRNAs expression has a wide variance in diverse types of cells and pathological states, and thus miR-modified exosome can change its functions. In addition, analyses have shown the high conservation of MiR-146a-5p amongst the mice, rats and humans. Other investigations also referred to the abundance of miR-146a-5p in the MSCs, and stimulatory impact of the humans’ umbilical cord MSC-exosome on the primordial follicles is applied by carrying the functional miR-146a-5p [230,231]. Accordingly, there can be an association between the advantageous functions of exosome and the increased levels of miR-146a-5p it carries. A number of investigations revealed that miR-146a protects against diverse brain impairment [232–234]. Moreover, it contributes to the regulation of microglia or macrophages in the ischemic stroke [235]. Additionally, miR-146a-5p downregulates in the ICH cases’ sera [236] and protects against ICH via suppressing TRAF6/NF-κB pathway [234]. Nonetheless, particular mechanism of miR-146a-5p in neuro-protection following the ICH should be highlighted.

Duan et al. addressed the impact of the exosome derived from the miR-146a-5p enriched bone-marrow mesenchymal stem-cells (BMSCs-miR-146a-5p-Exos) on the experimental ICH [237]. Analysis showed the induction of ICH in adult male Sprague-Dawley rats by intra-striatally injecting the collagenase type IV. Researchers verified binding miR-146a-5p and the respective target genes through the luciferase reporter assay. BMSCs-miR-146a-5p-Exos injection ameliorated the neurological functions, diminished degenerative and apoptotic neurons, and suppressed the inflammatory responses. Results also revealed the clear inhibition of M1 polarization of micro-glia after ICH in the rats by the miR-146a-5p enriched Exosome that has been followed by lower expression of the proinflammatory mediators released via M1 microglia-like monocyte chemo attractant protein-1 (MCP-1), the inducible nitric oxide synthase (iNOS), as well as cyclooxygenase-2 (COX-2). Consequently, direct targeting of interleukin-1 receptor--associated kinase1 (IRAK1) as well as nuclear factor of the activated T-cells 5 (NFAT5) has been shown by miR-146a-5p involved in inflammation responses and polarization of M1 microglia or macrophages. miR-146a-5p riched BMSCs-Exos afforded neuroprotection and functional improvement after ICH via reduction of neuronal apoptosis as well as inflammation related to suppression of the microglial M1 polarization via down-regulating IRAK1 and NFAT5expression [237].

Endogenous neural stem-cells of the adults’ brains have been largely found in the sub-ventricular zone (SVZ) area of lateral ventricle and sub-granular zone (SGZ) region of hippocampus [238,239]. Neural stem-cells are be activated following stroke and subsequently migrate into the lesioned areas and differentiate into the functional neural cells [240,241].

Exosomes from the peri-ischemic striatum and determined them through exosomal bio-markers so that the differentially expressed miRNAs have been detected with micro-array chip. In the next step, they cultured the primary stem cells and utilized OGD as well as reperfusion (OGD/R) for mimic vitro ischemic injuries. Analysis has shown the greater level of the exosomal bio-markers of CD81 and TSG101 in the peri-ischemic striatum following the EA treatment that displayed 25 differentially expressed miRNAs in the isolated exosomes, of which miR-146b has been chosen for additional analyses. Furthermore, EA enhanced the miR-146b expression and its suppressors have been capable of blocking the impacts. Researchers also approved that EA up-regulated miR-146b expression for promoting the differentiation of the neural stem cells into the neurons in the peri-ischemic striatum. *According to the results,* OGD/R delayed differentiating the neural stem-cells and thus miR-146b suppressors repressed its differentiation. Moreover, NeuroD1 showed its contribution to the differentiation of the neural stem cells into neurons. Additionally, in vivo experiments showed that EA enhanced differentiating the NeuroD1-mediated neural stem cells through miR-146b and ability of EA for improving the neurological deficiencies via miR-146b following the ischemic stroke. Finally, EA promoted differentiating the endogenous neural stem-cells through the exosomal miR-146b for improving the neurological injuries following the ischemic stroke [242].

miRNA-126 affords its neuro-protective effects against the ischemia injuries by regulating the genes expression like phosphor-inositide-3-kinase regulatory sub-unit 2 (PIK3R2). Moreover, the vascular cell adhesion molecule 1 (VCAM-1) in ECs [243,244]. VCAM-1 as well as PIK3R2 have a relationship to the resistance against endothelial dysfunction and vascular inflammation that have been proposed as the 2 prominent procedures correlated to the neuronal damages of reperfusion or ischemia [245]. Lower activation of the inflammatory pathways and endothelial dysfunctions must contribute to the RIPC-mediated neuro-protection through the exosomal miRNA-126 [246]. Chemical reagents such as DNMT inhibitors involve in the neuro-protection in the rodents that suffer from mild ischemia [247,248]. Pandi et al. [249] revealed that small interfering RNA (siRNA)-mediated DNMT3A knockdown decreased infarction in vivo as well as PC12-cell death in vitro. It is noteworthy that there is no information on the mechanisms involved in neuro-protection. Hu et al.[250]found that lower expression of DNMT may result in the greater expression level of a number of genes such as metallothionein because of hypo-methylation, protecting neuronal cells from the hypoxia damages or ischemia. Notably, DNMT3A andDNMT1 partially increased in SH-SY5Y cells that over-expressed miRNA-126 in our research. Consequently, a number of compensatory mechanisms possibly exist amongst DNMT3A, DNMT3B and DNMT1 so that one of them is lost or remarkably diminished [251].

Another investigation explored if exosomal miRNA-126 from RIPC serum may have neuro-protective role [252]. Researchers isolated the exosomes from the venous serum of 4 healthy-young male cases prior to and following RIPC. Consequently, the level of DNMT and DNMT3B activities has been down-regulated in the SH-SY5Y cells that have been incubated with the RIPC exosomes. Following the miRNA-126 over-expression in the SH-SY5Y cells, the overall methylation level as well as DNMT3B gene expression have been down-regulated in the above cells that has been compatible with the bio-informatics prediction. Finally, RIPC exosomes are capable of influencing the cell-cycle and increasing the tolerance of OGD in the SH-SY5Y cells and apparently RIPC has neuro-protective effects by down-regulating DNMTs expression in the neural cells via upregulating the serum exosomal miRNA-126 [252]. Table 3 lists exosomal miRNAs that are involved in stroke.

## 8. Exosomal long non-coding RNAs and stroke

Researchers showed the lncRNAs in exosomes [265]. Exosomal lncRNAs have shown as the attractive bio-markers for stroke and there is insufficient information on the contribution of exosomal lncRNAs to stroke pathogenesis.

Acute minor stroke (AMS) has been introduced as a sort of hypoxic ischemic necrosis with at least four National Institutes of Health Stroke Scale (NIHSS) score. Nonetheless, initial diagnosis of the AMS is difficult due to the absence of efficient molecular markers. Thus, experts in the field tended to reveal several long non-coding RNAs (lncRNAs) related to AMS.

Xu et al. examined potent bio-markers of lncRNAs in the exosomes isolated from the blood serum of AMS cases for initial detection of the disease [266]. For this reason, they utilized RNA-seq, GO enrichment and KEGG pathway analyses as well as RT-qPCR for validating the level of expression of 4 of 11 differentially expressed lncRNAs (lnc-NTRK3–4, lnc-CRKL-2, lnc-CALM1–7& RPS6KA2-AS1) that contribute to neuro-trophin signaling pathways. Moreover, expression level of lnc-NTRK3–4 and lnc-CRKL-2 considerably enhanced the cases suffering from AMS whereas level of expression of RPS6KA2-AS1 and lnc-CALM1–7 remarkably diminished. In conclusion, these newly revealed lncRNAs may be used as novel joint biomarkers for the early detection of AMS [266].

Chen et al. addressed modifications in the miRNAs and lncRNAs expression patterns in exosomes derived from the vascular endothelial cells upon heat stroke [267]. Analyses identified 10 considerably up-regulated and 10 down-regulated lncRNAs in exosomes derived from the heat stroke temperature treated cells. Consequently, KEGG (Kyoto Encyclopedia of Genes & Genomes) and GO (Gene Ontology) have been utilized for evaluating the signaling pathways of differential expression in lncRNAs. Interaction network of the lncRNAs-miRNAs-mRNA with ceRNA (competing endogenous RNA) principle revealed that the identified miRNAs and lncRNAs in the endothelial cell exosomes may be utilized as the noninvasive bio-markers for the heat strokes [267].

## 9. Conclusion

A variety of internal and external factors are associated with initiation and progression of stroke. Epigenetic regulations play essential tasks in the stroke pathogenesis. LncRNAs and miRNAs act as epigenetic regulators which are involved in the modulation of several biological mechanisms such as angiogenesis, growth, and differentiation. Several studies revealed that these molecules have critical roles in various stages of stroke. LncRNAs and miRNAs, as main regulators, have therapeutic, diagnostic and prognostic potential in the brain diseases including stroke. Circulating ncRNAs which have cell type- and tissue-specific expression patterns and remarkable stability in peripheral blood have also been studied to detect patients at risk for incident or recurrent stroke, and to predict stroke outcome. Moreover, exosomes have been regarded as the new materials with the prominent inter-cellular roles in the mediation of neuro-restorative events following strokes and neural injuries. Advancement in exosome therapy would benefit from, 1. Identifying cellular signals through which ischemic brain can influence quantity as well as volume of exosomes released by the brain parenchymal cells and remote organs, 2. Getting information of the influence of exosomal cargo on expressing the endogenous genes and proteins in the recipient cells of the damaged brains, 3. Delineating particular kinds of cells targeted by the brain parenchymal cell–derived exosomes, and 4. Deciphering the impacts of gender, comorbidity and age on cellular production of exosomes and the respective cargo as well as the impact of comorbidity, age and gender in responding to the exosome treatments after strokes. However, other investigations on exosomes as tools of inter-cellular communication in ischemic brain should provide in valuable information on the contribution of exosomes to stroke pathogenesis and exosomal therapy.

## Figures and Tables

**Table 1:** Selected lncRNAs involved in stroke pathogenesis.

LncRNA	Target (s)	Effect (s)	Study models	Ref
N1LR	Maybe inactivates p53	Inhibits neural death and apoptosis probably via reducing p53 phosphorylation	In vitro: N2a cells In vivo: SD rats, mice	[76]
MALAT1	E-selectin and Bim	Inhibits inflammation and endothelial cell death and defends cerebral parenchyma and microvasculature from brain ischemic injury.	In vitro: BMECs In vivo: C57BL/6 mice	[83]
H19	DUSP5-ERK1/2	Silenced autophagy which activated by OGD/R via DUSP5-ERK1/2 axis.	In vitro: SH-SY5Y cells In vivo: SD rats	[84]
TUG1	miR-9/Bcl2l11	Inhibits miR-9 and weakened suppression of bcl2l11 expression and so, leads a neurotoxicity after stroke.	In vitro: SH-SY5Y cells In vivo: SD rats	[85]
C2dat1	CaMKIIδ	Increases the CaMKIIδ expression and activates NF-κB signal impacting neuronal survival after ischemic stroke.	n vitro: N2a cells In vivo: C57BL/6 mice	[86]
ANRIL	VEGF/NF-κB	Increases VEGF expression and activates angiogenesis via activation of NF-κB pathway	In vivo: male Wistar rats	[87]

**Table 2:** Different microRNAs involving in stroke.

MicroRNA (s)	Expression status	Target (s)/Mechanism (s)	Study model	Ref
miR-296-5p	Down	CD73, PKN2	Mouse/MCAO	[195]
miR-686, miR-1224	Up	CD73, PKN2	Mouse/MCAO	[195]
miR-498, miR-25, miR-483-5p miR-34b	Down	BCL-2 modifying factor (BMF) and p53	Human/Ischemic stroke	[196]
miR-21	Up	SOD3, TNF-α	Rat/MCAO	[197]
mir-30a	Down	RhoB, beclin-1	Human	[198]
mir-126, mir-146	Up	VCAM-1, TLR4	Human	[199]
MiR-1259, miR-142-3p, miR-15b, miR-186, miR-519e, miR-768-5p	Down	Arl2	Human	[199]
miR-1184, Let-7e, miR-1246, miR-1261, miR-1275, miR-1285, miR-1290, miR-181a, miR-25, miR-513a-5p, miR-550, miR-602, miR-665, miR-891, miR-933, miR-923	Up	GPx1, NOX4	Human	[199]
miR-16, miR-23a, miR-150, miR-107, miR-185, miR-191, miR-292-5p, Let-7, miR-451, miR-327 and miR-494	Up	XIAP, APAF-1/caspase-9	Rat/MCAO	[200]
miR-26a, miR-26b, miR-29b, miR-140, miR-214, miR-328, miR-352, miR-320, miR-137	Down	NCX1	Rat/MCAO	[200]
miR-19b, miR-136, miR- 199a-3p, miR-32	Up	PI3K/Akt/mTOR signaling	Rat/MCAO	[201]
miR-290, miR-218, miR-133, miR-145	Up	caspase-9, -3 superoxide dismutase-2	Rat/MCAO	[202]
miR-27a, miR-204, miR-301, miR-338, miR-7, miR-137, miR-335, miR-148b, miR-98, miR-30e	Down	COXIV, SOD2, Txnrd2	Rat/MCAO	[202]
miR-155	Down	SHIP1, p53	Rat/MCAO	[203]
miR- 424	Up	Cullin 2, PU.1, RUNX-1 and C/EBPα	Mice/MCAO	[204]
miR-124	Up	VILIP-1	Rat/MCAO	[205, 206]

**Table 3:** Exosomal miRNAs in stroke.

Cargo (s) Detection methods	Effect (s)	References
miR-17–92	Increases neural plasticity and functional recovery after stroke, possibly via targeting phosphatase and tensin homolog.	[253]
MiR-133b	promotes neural plasticity and functional recovery after treatment of stroke	[254]
miR-133b	improve neural plasticity and functional recovery after stroke	[255]
MiR-30d-5p	Prevent cerebral injury by inhibiting autophagy-mediated microglial polarization to M1.	[256]
MiR-126	Promotes neurorestorative effects in T2DM mice.	[257]
miR-181c-3p	Inhibits Neuroinflammation by Downregulating CXCL1 in Astrocytes.	[258]
miR-138-5p	Reduces neurological impairment by promoting proliferation and inhibiting inflammatory responses of astrocytes following IS by targeting LCN2.	[259]
miR-124	Promotes Neurogenesis	[260]
microRNA-26a	Enhanced neurogenesis after ischemic stroke.	[261]
miR-146b	Improve neurological injury after ischemic stroke.	[242]
MicroRNA-17-92	Enhance Neuroplasticity and Functional Recovery After Stroke.	[262]
miR-146a-5p	Inhibiting Neuronal Apoptosis and Microglial M1 Polarization.	[237]
miR-124	Cortical neurogenesis is increased.	[263]
miR-134	It enhanced expressing LIM domain kinase to increase plasticity of synaptic-dendritic after Ischemic stroke.	[264]

## Abbreviations:

AGO: Argonaute
AIF: apoptosis-inducing factor
ANRIL: antisense non-coding RNA in the INK4 locus
APAF-1: Apoptotic protease activating factor-1
Arg-1: Arginase 1
Arl2: ADP Ribosylation Factor Like GTPase 2
BBB: blood-brain barrier
Bcl-2: B-cell lymphoma 2
BDNF: brain-derived neurotrophic factor
BMECs: brain microvascular endothelial cells
BMF: BCL-2 modifying factor
CaMKIIδ: Ca(2+)/calmodulin-dependent protein kinase II delta
CARD: Caspase recruitment domain family member
CD: Cluster of Differentiation
C2dat1: CaMK2D-associated transcript 1
CDKN2A: Cyclin Dependent Kinase Inhibitor 2A
CDKN2B: Cyclin Dependent Kinase Inhibitor 2B
C/EBP-α: CCAAT/enhancer-binding protein alpha
ChIRP-Seq: Chromatin Isolation by RNA purification
CLIP: crosslinking immunoprecipitation
CMPs: chromatin-modifying proteins
coREST: co-repressors of the transcription factor REST
CRISPR: clustered regularly interspaced short palindromic repeats
Cytc: cytochrome c
DGCR8: DiGeorge syndrome critical region 8
dsRNA: Double-strand RNA
DUSP5: Dual specificity phosphatase 5
EAE: experimental autoimmune encephalomyelitis
ERK: extracellular signal-regulated kinase
FasL: Fas ligand
FLT-1: Fms-like tyrosine kinase 1
FosDT: Fos downstream transcript
GluR2: glutamate receptor 2
GPx1: Glutathione Peroxidase 1
HIF-1α: Hypoxia-inducible factor 1-alpha
HMGB1: high mobility group box 1 protein
HO-1: hemeoxygenase-1
H2O2: hydrogen peroxide
ICH: Intracerebral hemorrhage
IGF-1: insulin-like growth factor-1
Igf2: Insulin Like Growth Factor 2
IL13Rα1: Interleukin 13 receptor, alpha 1
I/R: Ischemia/Reperfusion
IL: interleukin
IRAK4: interleukin-1 receptor-associated kinase 4
Limk1: LIM Domain Kinase 1
lncRNAs: long non-coding RNAs
MALAT1: Metastasis Associated Lung Adenocarcinoma Transcript 1
MCAO: middle cerebral artery occlusion
MCP-1: monocyte chemotactic protein-1
MEG3: maternally expressed gene 3
miRISC: miRNA-induced silencing complex
miRNAs: microRNAs
MnSOD: manganese SOD
Nck1: non-catalytic region of tyrosine kinase adaptor protein 1
ncRNAs: Non-coding RNAs
NCX: sodium-calcium exchanger
N2a: Neuro2a
NF-κB: nuclear factor kappa-light-chain-enhancer of activated B cells
NLRP3: NOD-, LRR- and pyrin domain-containing protein 3
NMDA: N-methyl-D-aspartic acid
nNOS: nitric oxide synthase
NOX4: NADPH Oxidase 4
NPCs: neural precursor cells
Nqo1: NAD(P)H Quinone Dehydrogenase 1
Nrf2: nuclear factor namely erythroid-2-related factor-2
NSCs: neural stem cells
OGD: oxygen-glucose deprivation
OGD/R: Oxygen-glucose deprivation and reperfusion
P-bodies: processing bodies
PI3K: Phosphoinositide 3-kinases
PKN2: Protein Kinase N2
Pol II: polymerase II
PRC1/2: polycomb repressive complex 1 and 2
pre-miRNAs: precursor miRNAs
pri-miRNAs: primary miRNAs
RACE: Rapid Amplification of cDNA Ends
RAGE: Receptor for advanced glycation end products
REST: repressor element-1 silencing transcription factor
RhoB: Ras Homolog Family Member B
RNAi: RNA interference
RNase P: ribonuclease P
RNA-seq: RNA sequencing
RNS: Reactive nitrogen species
ROS: reactive oxygen species
RUNX-1: Runt-related transcription factor 1
SHIP1: SH-2 containing inositol 5′ polyphosphatase 1
SNHG14: small nucleolar RNA host gene 14
snoRNA: small nucleolar RNA
snoRNP: small nucleolar ribonucleoprotein
SNP: single nucleotide polymorphism
SOD: superoxide dismutase
ssRNA: single-stranded RNA
TGF: transforming growth factor
TLRs: Toll-like receptors
TNF-α: Tumor necrosis factor alpha
TRBP: transactivation-responsive RNA-binding protein
TUG1: Taurine Up-Regulated 1
Txnrd2: Thioredoxin Reductase 2
VCAM-1: vascular cell adhesion molecule 1
VEGF: vascular endothelial growth factor
VILIP-1: Visinin like protein 1
WHO: World Health Organization
XIAP: X-linked inhibitor of apoptosis protein
XPO5: exportin 5
